# Personalised rehabilitation to improve return to work in patients with persistent spinal pain syndrome type II after spinal cord stimulation implantation: a study protocol for a 12-month randomised controlled trial—the OPERA study

**DOI:** 10.1186/s13063-022-06895-5

**Published:** 2022-12-05

**Authors:** Maarten Moens, Lisa Goudman, Dominique Van de Velde, Lode Godderis, Koen Putman, Jonas Callens, Olivia Lavreysen, Dries Ceulemans, Laurence Leysen, OPERA consortium, Jean-Pierre Van Buyten, Iris Smet, Ali Jerjir, Bart Bryon, Mark Plazier, Vincent Raymaekers, Sam Schelfout, Erwin Crombez, Tom Theys, Anaïs Van Hoylandt, Philippe De Vloo, Bart Nuttin, Lieven Annemans, Elien Van der Gucht, Anneleen Leyman, Ann De Smedt

**Affiliations:** 1 STIMULUS research group, Vrije Universiteit Brussel, Laarbeeklaan 103, Jette, 1090 Belgium; 2 Department of Neurosurgery, Universitair Ziekenhuis Brussel, Laarbeeklaan 101, Jette, 1090 Belgium; 3 Department of Radiology, Universitair Ziekenhuis Brussel, Laarbeeklaan 101, Jette, 1090 Belgium; 4 Center for Neurosciences (C4N), Vrije Universiteit Brussel, Laarbeeklaan 103, Jette, 1090 Belgium; 5 Pain in Motion Research Group (PAIN), Department of Physiotherapy, Human Physiology and Anatomy, Faculty of Physical Education & Physiotherapy, Vrije Universiteit Brussel, Laarbeeklaan 103, Jette, 1090 Belgium; 6 Research Foundation Flanders (FWO), Egmontstraat 5, Brussels, 1000 Belgium; 7 Faculty of Medicine and Healthcare Sciences, Department of Rehabilitation Sciences, Occupational Therapy Program, Ghent University, Ghent, 9000 Belgium; 8 Centre for Environment and Health, Department of Public Health and Primary Care, KU Leuven (University of Leuven), Leuven, 3000 Belgium; 9 IDEWE, External Service for Prevention and Protection at Work, Heverlee, 3001 Belgium; 10 Interuniversity Centre for Health Economics Research (I-CHER), Department of Public Health (GEWE), Faculty of Medicine and Pharmacy, Vrije Universiteit Brussel, Laarbeeklaan 103, Jette, 1090 Belgium; 11 Department of Physical Medicine and Rehabilitation, Universitair Ziekenhuis Brussel, Laarbeeklaan 101, Jette, 1090 Belgium

**Keywords:** Rehabilitation, Failed back surgery syndrome, Neuromodulation, Personalised medicine, Randomised controlled trial

## Abstract

**Background:**

For patients with therapy-refractory persistent spinal pain syndrome type II (PSPS-T2), spinal cord stimulation (SCS) may serve as an effective minimally invasive treatment. Despite the evidence that SCS can improve return to work (RTW), only 9.5 to 14% of patients implanted with SCS are effectively capable of returning to work. Thus, it seems that current post-operative interventions are not effective for achieving RTW after SCS implantation in clinical practice. The current objective is to examine whether a personalised biopsychosocial rehabilitation programme specifically targeting RTW alters the work ability in PSPS-T2 patients after SCS implantation compared to usual care.

**Methods:**

A two-arm, parallel-group multicentre randomised controlled trial will be conducted including 112 patients who will be randomised (1:1) to either (a) a personalised biopsychosocial RTW rehabilitation programme of 14 weeks or (b) a usual care arm, both with a follow-up period until 12 months after the intervention. The primary outcome is work ability. The secondary outcomes are work status and participation, pain intensity, health-related quality of life, physical activity and functional disability, functional capacities, sleep quality, kinesiophobia, self-management, anxiety, depression and healthcare expenditure.

**Discussion:**

Within the OPERA project, we propose a multidisciplinary personalised biopsychosocial rehabilitation programme specifically targeting RTW for patients implanted with SCS, to tackle the high socio-economic burden of patients that are not re-entering the labour market. The awareness is growing that the burden of PSPS-T2 on our society is expected to increase over time due to the annual increase of spinal surgeries. However, innovative and methodologically rigorous trials exploring the potential to decrease the socio-economic burden when patients initiate a trajectory with SCS are essentially lacking.

**Trial registration:**

ClinicalTrials.gov NCT05269212. Registered on 7 March 2022.

## Administrative information

Note: the numbers in curly brackets in this protocol refer to SPIRIT checklist item numbers. The order of the items has been modified to group similar items (see http://www.equator-network.org/reporting-guidelines/spirit-2013-statement-defining-standard-protocol-items-for-clinical-trials/).

**Table:** 

Title {1}	Personalised rehabilitation to improve return to work in patients with Persistent Spinal Pain Syndrome Type II after Spinal Cord Stimulation implantation: a study protocol for a 12-month randomised controlled trial – the OPERA study
Trial registration {2a and 2b}.	ClinicalTrials.gov NCT05269212. Registered on 7 March 2022.
Protocol version {3}	Protocol Version 3, May 2022.
Funding {4}	This study is funded by Research Foundation Flanders (FWO), Belgium (project number T000821N).
Author details {5a}	Maarten Moens^1–5^, Lisa Goudman^1, 2, 4–6^, Dominique Van de Velde^7^, Lode Godderis^8, 9^, Koen Putman^10^, Jonas Callens^1, 10^, Olivia Lavreysen^8^, Dries Ceulemans^7^, Laurence Leysen^1, 5^, OPERA consortium, Ann De Smedt^1, 4, 11^. ^1^STIMULUS research group, Vrije Universiteit Brussel, Laarbeeklaan 103, 1090 Jette, Belgium. ^2^Department of Neurosurgery, Universitair Ziekenhuis Brussel, Laarbeeklaan 101, 1090 Jette, Belgium. ^3^Department of Radiology, Universitair Ziekenhuis Brussel, Laarbeeklaan 101, 1090 Jette, Belgium. ^4^Center for Neurosciences (C4N), Vrije Universiteit Brussel, Laarbeeklaan 103, 1090 Jette, Belgium. ^5^Pain in Motion Research Group (PAIN), Department of Physiotherapy, Human Physiology and Anatomy, Faculty of Physical Education & Physiotherapy, Vrije Universiteit Brussel, Laarbeeklaan 103, 1090 Jette, Belgium, www.paininmotion.be. ^6^Research Foundation Flanders (FWO), Egmontstraat 5, 1000 Brussel, Belgium. ^7^Faculty of Medicine and Healthcare Sciences, Department of Rehabilitation Sciences, Occupational Therapy Program, Ghent University, 9000 Ghent, Belgium. ^8^Centre for Environment and Health, Department of Public Health and Primary Care, KU Leuven (University of Leuven), 3000 Leuven, Belgium. ^9^IDEWE, External Service for Prevention and Protection at Work, 3001 Heverlee, Belgium. ^10^Interuniversity Centre for Health Economics Research (I-CHER), Department of Public Health (GEWE), Faculty of Medicine and Pharmacy, Vrije Universiteit Brussel, Laarbeeklaan 103, 1090 Jette, Belgium. ^11^Department of Physical Medicine and Rehabilitation, Universitair Ziekenhuis Brussel, Laarbeeklaan 101, 1090 Jette, Belgium.
Name and contact information for the trial sponsor {5b}	Vrije Universiteit Brussel, Maarten Moens, maarten.TA.moens@vub.be
Role of sponsor {5c}	The funders had no influence on the research reported in this paper.

## Introduction

### Background and rationale {6a}

The incidence of patients that will develop persistent spinal pain syndrome type II (PSPS-T2) after previous spinal surgery, formerly known as failed back surgery syndrome, is estimated in the range of 10–40%, dependent on the exact type of surgery [1]. This heterogeneous group of patients with PSPS-T2 suffers from persistent or recurring low back pain despite having undergone lumbosacral spine surgery (of any type), sometimes associated with referred or radiating leg pain [2]. For therapy-refractory PSPS-T2, meaning conservative treatment did not achieve adequate pain relief, spinal cord stimulation (SCS) may serve as a minimally invasive treatment option with proven cost-effectiveness [3]. The aim of SCS is to make chronic pain tolerable, by achieving substantial pain relief and improvements on several components among which disability, medication use, health-related quality of life and psychological status [4, 5].

Recently, awareness has increased concerning the tremendous influence of chronic pain on work absenteeism [6]. For patients with chronic pain, work resumption could enable social interactions, increase self-esteem, facilitate social participation, ensure financial security and optimise psychological well-being [7, 8]. Despite those benefits, the number of patients treated with SCS that eventually returns to work is rather limited. A meta-analysis previously indicated that SCS proved to be an effective approach to stimulate return to work (RTW) in patients with specific chronic pain syndromes [9]. More specifically, the odds of being at work (OR 2.15; 95% CI 1.44–3.21; *p*<0.001) and returning to work (OR 29.06; 95% CI 9.73–86.75; *p*<0.001) were higher in patients post-SCS compared to before SCS. Despite the favourable results that SCS is an effective approach to stimulate RTW, in absolute numbers, only 9.5 to 14% of patients implanted with SCS are effectively returning to work [9–11]. Therefore, it seems that current post-operative interventions are not effective to achieve RTW after SCS implantation.

In patients with chronic low back pain without previous spinal surgery, multidisciplinary biopsychosocial rehabilitation programmes proved to be beneficial to promote work resumption [12, 13]. For patients with persisting or recurring pain after previous spinal surgery, who started with SCS as a pain management strategy, descriptions of rehabilitation programmes and concrete paramedical therapy guidelines to improve RTW are scarce. Physical therapy guidelines after SCS implantation promote a patient-centred approach to address individual needs, values and goals with special attention for the risks of lead migration or fracture and/or damage to SCS componentry [14]. The lack of high-intensive RTW rehabilitation in this group presumably leads to the low percentage of patients that returns to their previous professional activities. Therefore, we propose a personalised biopsychosocial rehabilitation programme to improve RTW for patients with PSPS-T2, implanted with SCS.

### Objectives {7}

The aim of this study is to examine whether the work ability in PSPS-T2 patients after SCS implantation is different with a personalised biopsychosocial rehabilitation programme specifically targeting RTW, compared to usual care. The secondary objective of the study is to examine if a personalised biopsychosocial rehabilitation programme specifically targeting RTW, compared to usual care, is different in improving sleep quality, work status and participation; obtaining pain relief; increasing health-related quality of life, physical activity, functional capacity and self-management; and decreasing functional disability, kinesiophobia, anxiety, depression and healthcare expenditure.

### Trial design {8}

OPERA is a two-arm, parallel-group multicentre randomised controlled trial evaluating whether the work ability in PSPS-T2 patients after SCS implantation is different after a personalised biopsychosocial rehabilitation programme specifically targeting RTW, compared to usual care (difference design). Patients will be randomised (1:1) to (a) a personalised biopsychosocial RTW rehabilitation programme of 14 weeks or (b) a usual care arm, both with a follow-up period of 12 months after the intervention.

## Methods: participants, interventions and outcomes

### Study setting {9}

The study will be conducted in three academic hospitals (Universitair Ziekenhuis Brussel, Universitair Ziekenhuis Gent, Universitair Ziekenhuis Leuven) and three regional (non-academic) hospitals (AZ Turnhout, Vitaz, Jessa Ziekenhuis). All study sites are located in Belgium. Details on study sites can be found at ClinicalTrials.gov with identifier: NCT05269212, 7th of March 2022.

### Eligibility criteria {10}

This study will focus on patients with chronic back and leg pain (due to PSPS-T2) who previously underwent spinal surgery and are scheduled for SCS implantation. Inclusion criteria are:

- Being diagnosed with PSPS-T2 (defined as patients suffering from neuropathic pain of radicular origin with pain in the lower back and/or leg(s), of an intensity of at least 4/10 on the Numeric Rating Scale, for a period of at least 6 months after a minimum of one anatomically successful spinal surgery and being refractory to conservative treatment (according to Belgian reimbursement rules from January 1, 2018))
- Being scheduled for SCS implantation
- Being between the age of 18 and 60 years (to be able to reintegrate in labour market)
- Being able to read, write and speak Dutch

Exclusion criteria are pregnancy and suffering from another chronic illness characterised by generalised widespread pain (e.g. rheumatoid arthritis, fibromyalgia, chronic fatigue syndrome, scleroderma).

### Who will take informed consent? {26a}

The treating neurosurgeon or anaesthesiologist (local principal investigator or his/her designee) will inform eligible patients about the project. Thereafter, an investigator of the OPERA consortium will contact the patients by telephone to further inform eligible patients about the project. In case they provide oral consent, they will be screened for inclusion and exclusion criteria as listed above during this telephone call. Patients who are eligible for participation (based on the telephone interview) and are willing to participate will receive detailed oral and written information about the study and have the opportunity to ask questions. Subsequently, they will be asked to provide written informed consent before participation.

### Additional consent provisions for collection and use of participant data and biological specimens {26b}

There are no planned ancillary studies involving the collection or derivation of data for purposes that are separate from the main trial. No biological samples will be obtained in this study.

## Interventions

### Explanation for the choice of comparators {6b}

Programmes incorporating the biopsychosocial approach into rehabilitation programmes for low back pain, thereby addressing physical deconditioning, pain coping mechanisms and workplace and health system barriers, revealed a positive effect on either RTW or reduction of the number of sick-leave days [15, 16]. In general, strength and endurance training [12, 17], behavioural interventions [18, 19] and specific vocational training (which includes workplace interventions and participatory ergonomics, if necessary [20]) are key components of these programmes. Due to the limited number of patients with PSPS-T2 that is able to RTW after SCS implantation in Belgium, a personalised biopsychosocial rehabilitation programme of 14 weeks to improve RTW was developed. This new treatment will be compared to the usual care that is currently provided to patients after SCS implantation.

### Intervention description {11a}

#### Experimental intervention: a personalised biopsychosocial RTW programme

The experimental intervention consists of a personalised biopsychosocial RTW programme, which will be provided at each centre. This 14-week programme will start 6 weeks (±1week) after implantable pulse generator (IPG) implantation and comprises 30 treatment sessions, delivered by trained physiotherapists, occupational therapists and psychologists. All therapists will be rigorously trained by experts in the different therapy modalities through a training session (half day) consisting of general project information as well as a specific training for the intervention that the therapist will deliver (i.e. physiotherapy, occupational therapy or pain neuroscience/RTW education). Each therapist will receive a written handbook with a detailed description of the content of each interventional session, as well as a checklist of topics that have to be covered per session and accompanying slides per session (except for physiotherapy sessions no slides are foreseen since the sessions only consist of exercises). Refreshment courses will be foreseen for all therapists (at least on an annual basis). Patients receive a written booklet with the content of the pain neuroscience education, a summary of the occupational sessions and home exercises for physiotherapy.

The programme starts with educational sessions regarding pain neuroscience and the key attributes of a RTW programme and nutritional advice. This will be followed by a session to determine individual treatment goals. The IMBA methodology (Integration von Menschen mit Behinderungen in die Arbeitswelt) [21] will be used to compare work requirements and work ability, while a functional capacity evaluation will be used to select relevant treatment sub-goals for the physical therapy and occupational therapy with the aim of being physically capable to work. As such, a standardised treatment plan, based on the type of job and the functional capacity of each patient, will be constructed with specific goals for both the physiotherapist and occupational therapist. To monitor treatment progress and enable adjustments to the rehabilitation plan, a shortened version of the functional capacity evaluation will be assessed at the beginning of week 8. Table 1 presents an overview of the content of the experimental intervention.

**Table 1:**
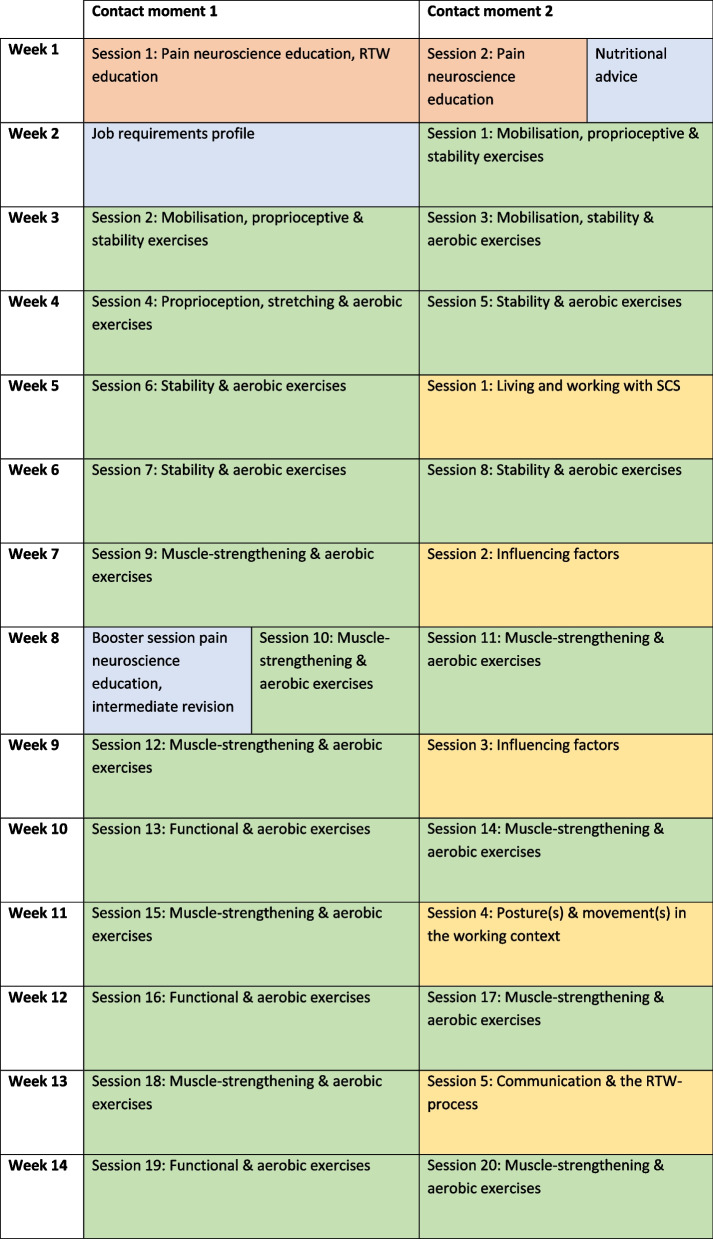
Organisation of the therapeutic sessions in the rehabilitation programme to improve return to work

Psychologist: orange-shaded cells, physiotherapist: green-shaded cells, occupational therapist: yellow-shaded cells, researcher: blue-shaded cells

*Abbreviation: RTW* return to work

##### Education about pain and RTW (first week + remotely + week 8 of the rehabilitation programme) 

Pain neuroscience education will be applied to reconceptualise pain and to bring awareness that pain is an output of the brain and that hypersensitivity of the central nervous system rather than local tissue damage contributes to their symptoms [22].

Patients will participate in an initial group session (maximum 6 persons/group) in which they will be educated about pain neuroscience and the key attributes of a RTW programme. This session will last about 1.5 h and includes the possibility for patients to ask questions during the session. After this first session, patients receive the information booklet as a refresher and tool to inform the significant other. Besides that, patients will have access to online coaching content (educational web application, available for free at https://www.retrainpain.org/) with a rehearsal of the content of the pain neuroscience education, and additional information about the influence of lifestyle factors. The second session during the first week is a 30-min individual session addressing specific questions and translating the content to the daily life of the patient. Finally, a follow-up session will be provided at the beginning of week 8 of the intervention programme to further explore individual patient cognitions and applications of pain neuroscience education into activities of daily living.

The content, format and pictures of the educational sessions are based on the books *Explain Pain* and *Pijneducatie een praktische handleiding voor (para)medici*, as used in previous research [23]. The ‘neuroscience education’ covers the physiology of the nervous system in general and of the pain system in particular. Topics addressed will include the characteristics of acute versus chronic pain, how pain becomes chronic (plasticity of the nervous system, modulation, modification, central sensitisation, etc.) and potential sustaining factors of central sensitisation like emotions, stress, pain cognitions and pain behaviour. Pain neuroscience education can reduce pain, improve patient knowledge of pain [24], improve function and lower disability, reduce the influence of underlying psychosocial factors and reduce healthcare utilisation in patients with chronic musculoskeletal disorders [25].

The ‘RTW education’ covers the cognitive, physical and behavioural items that are considered to be the core items in an RTW process. The topics addressed in this session are based on the ICF core set ‘vocational rehabilitation’ and include information about intrinsic energy and drive functions, cognitive functions, exercise tolerance, skill-acquiring, handling stress and social support (by family, employer, health services, labour and employment services) [26].

To ascertain the quality of the pain neuroscience education, all patients (*N*=112) will be asked to complete the Neurophysiology of Pain Test before and after the full intervention (experimental or control). The educational session is developed in line with the content of the Dutch Neurophysiology of Pain Test, in such a way that after having received the education, patients of the experimental intervention should be able to answer the questions of the test correctly [27].

##### Nutritional advice (week 1 of the rehabilitation programme) 

A positive association was found between excess weight/obesity and low back pain [28]. Low back pain intensity and disability show dose responses to body mass index, waist circumference, fat percentage and fat mass [29]. Nutritional interventions, especially an altered dietary pattern and altered intake of specific nutrients, result in significant pain relief in patients with chronic pain [30]. Therefore, increasing recommendations are put forward to target these aspects in chronic pain patients [31]. An online session of nutritional advice is implemented in the rehabilitation programme to create awareness, provide advice and discuss potential issues in order to facilitate adopting a healthier diet.

##### Goal setting session based on functional capacity evaluation (week 2+8 of the rehabilitation programme)

Getting a clear view on the working abilities of the patient by means of a functional capacity evaluation has been proven to be an indispensable step in the RTW process [32]. Functional capacity evaluations are performance-based batteries of tests, designed to observe the functional capacity and, based on this, to determine the individual’s ability to cope with the physical demands of work [33]. In these tests, work-related activities such as kneeling, walking, lifting and carrying are systematically evaluated. Patients are estimated as being able to safely return to their work, if their physical abilities are at least at the same level of what is required at work [34]. After this assessment, performed 6 weeks after IPG implantation (i.e. just before the start of the rehabilitation programme), it is necessary to interpret these findings in relation to the specific demands from the job. Therefore, the functional capacity evaluation will be used in combination with the IMBA methodology. Clear associations have been found between the IMBA, the Dictionary of Occupational Titles (DOT, 1991) and RTW wherein being able to crouch was an important predictor for short-term and long-term employment outcomes [35]. The result is a report in which the physical capacity is visually compared to the demands from the job. This report will enable the construction of sub-goals aiming to deliver an individualised, but standardised physical and occupational therapy programme. A personalised approach will be used, thereby optimally preparing the patient for his/her specific job.

A shortened functional capacity evaluation, only comprising relevant work requirement items based on comparisons according to the IMBA methodology, will be re-evaluated at the beginning of week 8, to monitor treatment progress and enable adjustments to the rehabilitation plan.

##### Physical therapy training (weeks 2–14 of the rehabilitation programme)

Once adaptive beliefs about chronic pain and exercise are acquired, a time-contingent approach will be applied in order to deactivate brain-orchestrated top-down pain facilitatory pathways [36]. The physiotherapist will individually adapt the exercises according to the level of physical capacities, psychosocial impairments, expectations and priorities of the patient [12]. Sessions will entail cardiorespiratory fitness, muscular strength, muscular flexibility, stabilisation exercises and proprioception training, all individually adapted [12, 37]. In the first phase, cognition-targeted motor control training will specifically focus on proprioception, coordination and sensorimotor control based on the idea of Sahrmann [38]. In combination with the acquired pain neuroscience principles, this time-contingent approach will entail exercising with a focus on retraining the deep muscles surrounding the lumbopelvic region [39]. In the second phase, more dynamic and functional exercises will be incorporated, combined with cardiorespiratory exercises through graded activity [40]. All physiotherapy sessions will last for 1 h and can be provided as group sessions.

##### Vocational therapy training programme (weeks 5, 7, 9, 11, and 13 of the rehabilitation programme)

The main focus of vocational training will be placed on physically difficult professional and daily living situations [12]. General needs (e.g. carrying, lifting, sitting) will be addressed according to the specific job demands of each patient [41]. Based on the physical abilities in the functional capacity evaluation, training will be provided on potential difficulties and/or problems in functional movements that are relevant for the job requirements. Additionally, living and working with a spinal cord stimulator, sleep advice, day-and-night rhythm and communication related to the work setting will be discussed with the patient on an individual basis during these sessions.

##### Informal contact with the occupational physician

Besides this physical rehabilitation programme, patients will be encouraged to return to their work. Occupational physicians will be involved from the start of the 14-week rehabilitation trajectory. More specifically, during the first weeks of the rehabilitation programme, a letter will be sent to the occupational physician of each patient with the physical health status of the patient and the specific rehabilitation goals. As such, the occupational physician is informed about the individual trajectory of the patient. For patients who are self-employed or not-employed, the general practitioner will receive this letter. Additionally, patients will be encouraged to have an informal meeting with their occupational physician to discuss the possibilities for RTW. By using an informal format, the occupational physician is not obliged to make any decisions, wherefore patients do not see these contact moments as a possible threat (e.g. professional confidentiality, difference with a ‘control doctor’). Within this project, we aim to develop a continuous communication (i.e. safe exchange of information) to ensure that the patient has all possible tools to enable RTW.

#### Control intervention: usual care

Patients randomly allocated to the control intervention will undergo the usual care trajectory. This programme is delivered at each of the participating centres. Patients will follow the usual care as it is implemented in Belgian hospitals, after SCS implantation. After implantation, patients are seen by the treating physician, pain nurse(s) and/or delegates of the companies of the SCS devices to programme the SCS parameters. On top of that, they have a fixed 6-month follow-up appointment to re-evaluate the therapy and evaluate medication use in combination with SCS. Each hospital can continue the usual care as normally provided to patients after SCS implantation. The normal trajectory of re-integrating patients will be followed and recorded.

### Criteria for discontinuing or modifying allocated interventions {11b}

The participants can withdraw from the study at any time.

### Strategies to improve adherence to interventions {11c}

Unblinded researchers will schedule all appointments between patients and therapists, according to the preferences of both parties. Patients will receive email notifications with an overview of their appointments. To track patient adherence, the ratio of the number of sessions that were followed versus the number of planned sessions will be calculated. Compliance will be calculated as the ratio of the total training intensity multiplied by the duration versus the prescribed total training intensity multiplied by the duration, afterwards multiplied by 100. The duration of each session will be written down by the patients in the information booklet at the end of each session.

Fidelity (i.e. the extent to which delivery of an intervention adheres to the protocol originally developed [42]) will be monitored by unblinded researchers through random on-site monitoring visits to evaluate the quality of the provided therapy. Quality will be evaluated with the aid of newly developed fidelity measurement checklists specifically focusing on the content of this rehabilitation programme, following ‘The Field Guide to Fidelity’ [43]. Separate checklists were constructed for both pain neuroscience education sessions, occupational therapy sessions and physiotherapy sessions, whereby 10% of each of the session types will be monitored throughout the duration of the project. After a monitoring, the researcher will discuss the quality of the session together with the therapist, based on the ratings on the checklist.

### Relevant concomitant care permitted or prohibited during the trial {11d}

Patients randomised to the rehabilitation programme will be asked not to start any new paramedical interventions between the start of the study and the end of the intervention, for example not initiating additional psychological interventions or starting with aerobic activities outside the rehabilitation programme. By imposing these restrictions, adherence to the protocol (i.e. 6-week rest, whereafter the rehabilitation programme will start) can be guaranteed.

### Provisions for post-trial care {30}

This study does not provide post-trial care. There is no anticipated harm for trial participation and consequently no compensation for anticipated harm.

### Outcomes {12}

**Fig. 1:**
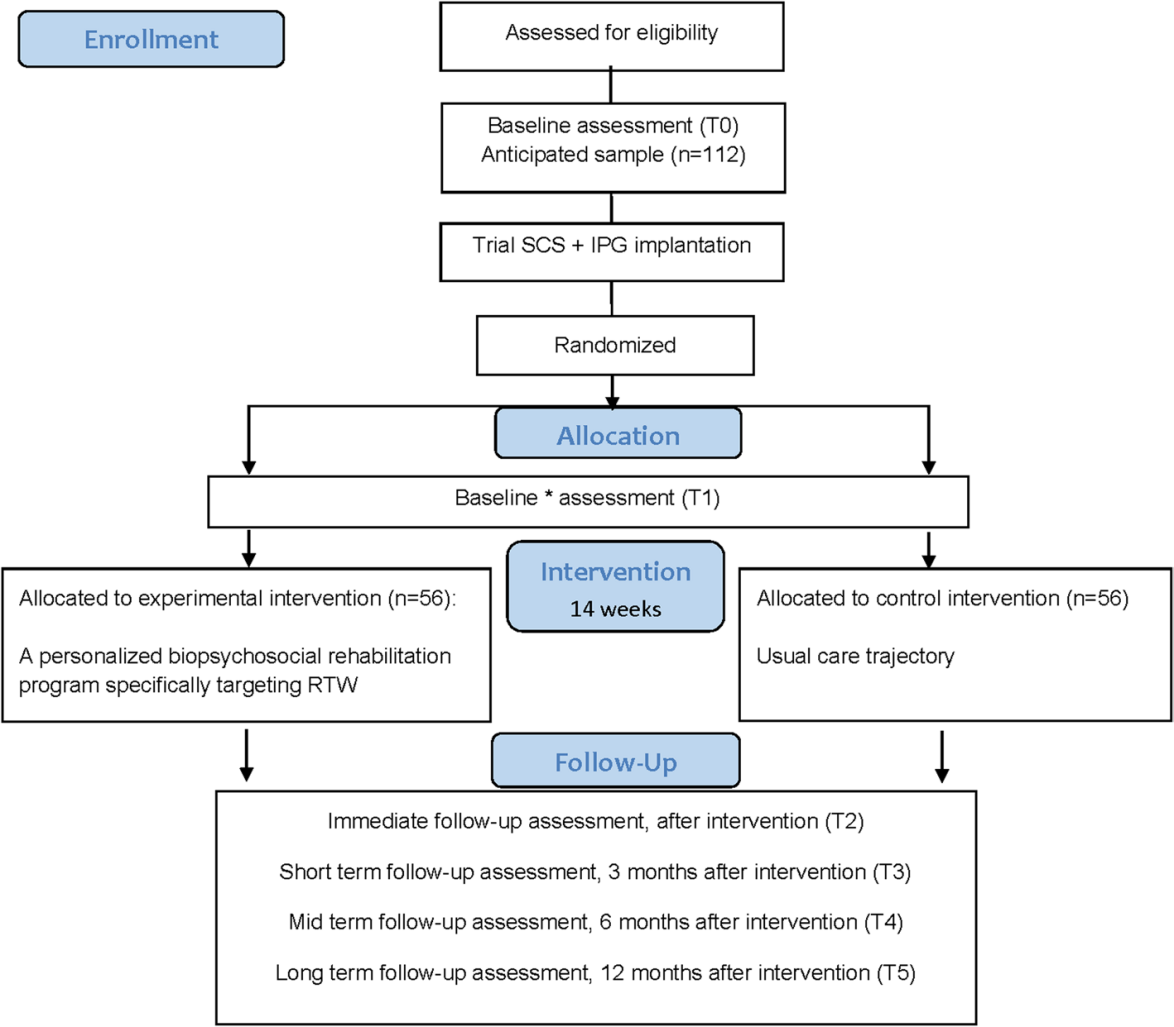
Project flowchart. Participant timeline. Abbreviations. IPG implantable pulse generator, *n* number, RTW return to work, SCS spinal cord stimulation, T time

At baseline, all outcomes will be evaluated. Afterwards, patients undergo a SCS trial period, followed by IPG implantation in case of a successful trial period (50% pain reduction and 50% reduction in pain medication use, according to the current Belgium reimbursement rules). Six weeks after the IPG implantation, the second assessment will take place. Follow-up assessments will be performed directly after the intervention of 14 weeks (immediate effects, short-term primary endpoint) and 3 months (short-term effects), 6 months (mid-term effects) and 12 months (long-term effects, long-term primary endpoint) after the intervention. The assessment directly after the intervention of 14 weeks will serve as the short-term primary endpoint, while the assessment at 12 months after the intervention serves as the long-term primary endpoint. To prevent test order effects, the test order of the self-reported measures will be randomised for each individual patient, at each assessment. At the 3-month visit, no actigraphy will be registered. During the other follow-up visit, all outcome measurements will be evaluated. Figure 1 presents the project flowchart.

#### Primary outcome

The ‘Work Ability Index’ (WAI) will be used as the primary outcome measure, which measures the work ability taking into account physical and mental parts of work as well as different diseases and their impact on work ability [44]. More specifically, the WAI consists of a questionnaire with 10 questions concerning current work ability compared with the lifetime best, work ability in relation to the job demands, number of current diseases diagnosed by a physician, estimated work impairment due to diseases, sick leave, self-prognosis of work ability and mental resources [45]. The total score of the WAI has a range from 7 to 49 with higher scores indicating higher work ability. This total score can further on be divided into four categories of work ability: poor (score from 7 to 27), moderate (score from 28 to 36), good (score from 3 to 43) and excellent (score from 44 to 49) [46].

#### Secondary outcome measurements

##### Functional capacity evaluation 

Functional capacity evaluations are commonly used for making fitness-for-work decisions and facilitating the RTW process [47]. An objective evaluation of the functional capacity will be performed by a shortened version of the Isernhagen Work Systems (now called WorkWell) functional capacity evaluation protocol. This functional capacity evaluation tool has already been used in chronic low back pain patients with acceptable test-retest reliability [48]. For this specific group of patients, a set of functional assessments that covers the seven basic postures and three lifting tasks will be applied. More specifically, the following tests are performed: standing tolerance, sitting tolerance, kneeling, crouching, forward bending, carrying, stair walking, repetitive sideways reaching, pulling, pushing, lifting low, lifting high and the 6-min walking test.

##### Work status and participation

The Work Rehabilitation Questionnaire (WORQ) will be used to measure work status and participation, which is a reliable questionnaire to evaluate functioning in vocational rehabilitation, based on the International Classification of Functioning, Disability and Health [49]. The information gained when using WORQ contributes to improving interdisciplinary understanding of the patient’s situation and therefore supports the integrative planning of the RTW process or engagement in gainful employment [50].

##### Pain intensity

The visual analogue scale (VAS-100 mm) will be used for the assessment of overall pain, defined as a combination of back and leg pain (but not pain from other body parts). The VAS pain score is believed to be reliable, valid and sensitive to change [51, 52].

##### Health-related quality of life

To describe the health-related quality of life, the EuroQol with 5 dimensions and 5 levels (EQ5D-5L) will be used [53]. Patients subjectively tick the box of the most appropriate statement in each of the 5 dimensions namely mobility, self-care, usual activities, pain/discomfort and anxiety/depression. The EQ5D-5L index scores range from −0.42 to 1, with 0 and 1 corresponding respectively to death and full health, based on preference-weighted health state classification algorithms [54]. Negative values denote health states perceived worse than death. Belgian population norms are available for the EQ5D-5L [55].

##### Physical activity and functional disability

In line with the IMMPACT recommendations for the assessment of physical activity in chronic pain clinical trials [56], actigraphy will be used to objectively capture continuous physical activity and rest/activity cycles in all patients [57]. Recordings will be made by an Actigraph device (ActiGraph, Pensacola, FL, USA). This device will be worn for 1 week after each study visit (except for the 3-month visit).

The Oswestry disability index (ODI) is used to measure functional disability due to abnormalities of the spine [58]. It contains ten topics whereby each topic is scored on a scale from 0 (no disability) to 5 (maximum disability possible). Higher values represent more disability.

##### Sleep quality

Perceived sleep quality will be measured with the Pittsburgh Sleep Quality Index (PSQI). The PSQI is by far the most widely used assessment of subjective sleep quality and contains 7 different sleep-related components: sleep quality, latency, duration, habitual sleep efficiency, sleep disturbance, use of hypnotics and daytime functioning [59].

##### Kinesiophobia

The Tampa Scale for Kinesiophobia (TSK) consists of 17 items to evaluate kinesiophobia in patients with low back pain [60]. Higher scores indicate a higher degree of kinesiophobia. The clinimetric properties are well-established in patients with low back pain [61].

##### Healthcare expenditure

The intervention is expected to have consequences on health-seeking behaviour, based on a previous reporting of pain education in low back pain patients [62]. As patients will learn how to handle their pain better, it is believed that their outreach for healthcare services will be lower with maintaining their quality of life. This will be measured by self-reporting methods (week diaries during the intervention, questionnaires at all other visits). Hence, healthcare expenditure includes hospitalizations and any kind of post-SCS treatment and consultations (e.g. pain killers, physiotherapy, psychotherapy, osteopathy).

##### Anxiety and depression

The Hospital Anxiety and Depression Scale (HADS) aims to measure symptoms of anxiety and depression and consists of 14 items: seven items for the anxiety subscale (HADS Anxiety) and seven for the depression subscale (HADS Depression). Each item is scored on a response scale with four alternatives ranging between 0 and 3. After adjusting for six items that are reversed scored, all responses are summed to obtain the two subscales. Recommended cut-off scores are 8–10 for doubtful cases and ≥ 11 for definite cases [63]. The HADS was found to perform well in assessing the symptom severity of anxiety disorders and depression in both somatic, psychiatric and primary care patients and in the general population [64].

##### Patient activation and self-management

Patient activation measure-13 (PAM) is a 13-item instrument to assess self-reported behaviour, knowledge and confidence for self-management of one’s health. The PAM has proven to be a reliable instrument to measure patient activation and self-management [65]. Patients will be divided in 4 levels, going from disengaged with a lack of confidence (level 1) to individuals who maintain their healthy lifestyle and feel confident about their health (level 4).

### Participant timeline {13}

**Fig. 2:**
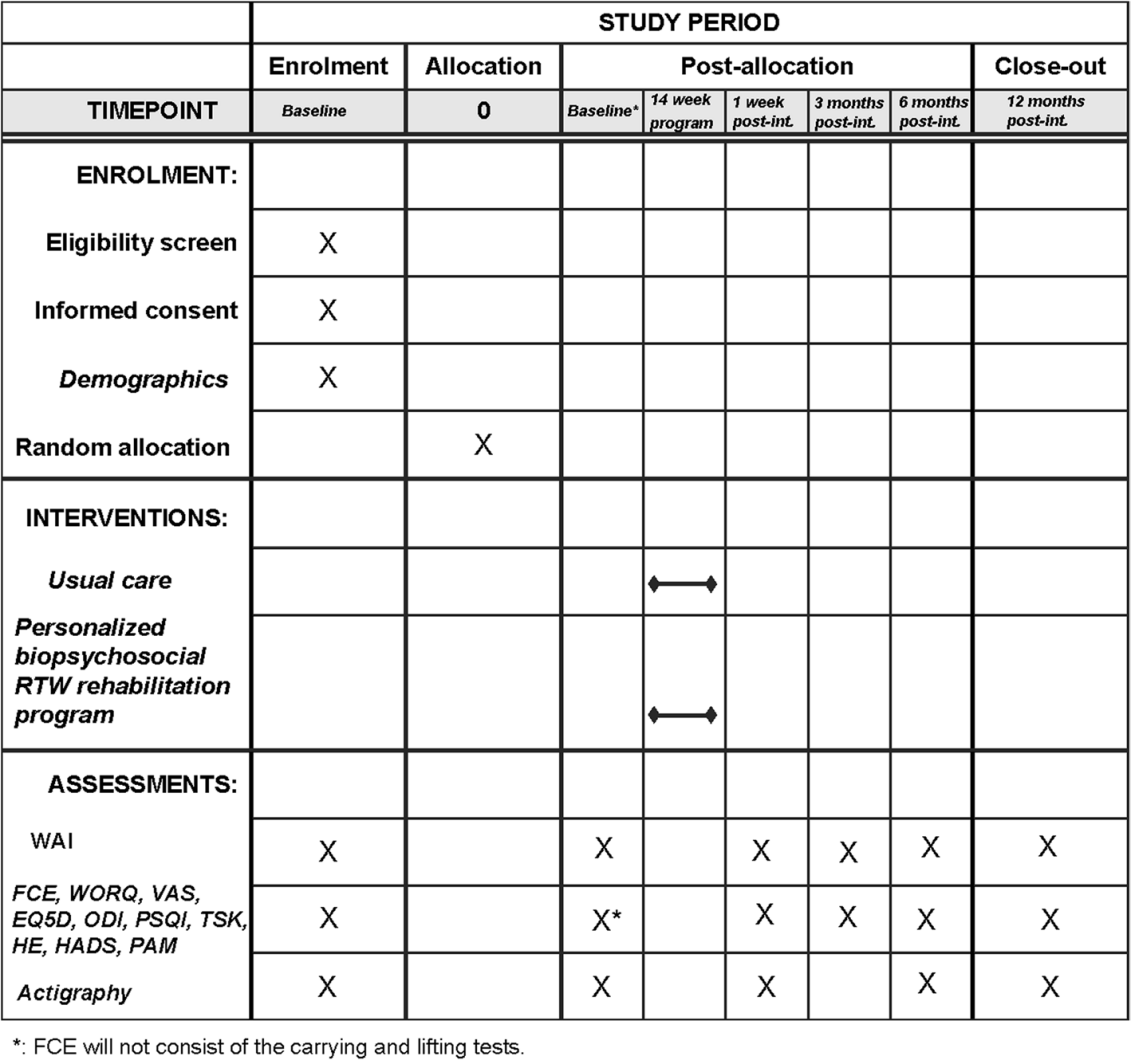
Participant timeline (SPIRIT). Abbreviations: EQ5D EuroQol with five dimensions and 5 levels, FCE functional capacity evaluation, HADS Hospital Anxiety and Depression Scale, HE healthcare expenditure, ODI Oswestry disability index, PAM patient activation measure-13, post-int post-intervention, PSQI Pittsburgh Sleep Quality Index, RTW return to work, TSK Tampa Scale for Kinesiophobia, VAS visual analogue scale, WAI Work Ability Index, WORQ Work Rehabilitation Questionnaire

The participant timeline for OPERA is presented in Fig. 2.

### Sample size {14}

Sample size calculation was performed using G*Power version 3.1.9.2 for evaluating differences in work ability between both interventions (the control and experimental intervention). Based on the least favourable design (two treatment arms, in which patients are measured three times) with a small effect size (*η*^2^=0.069; *d*=0.28) [66], a sample size of 112 patients is needed to reach a significant between-subject effect. This sample size calculation accounts for a 20% loss to follow-up after 1 year and for a SCS trial failure rate of 11.3%, according to Belgian reimbursement rules [5]. Calculations were based on two-tailed testing (alpha=0.05) with 85% power. Allocation ratio (N2/N1) was defined as 1, resulting in 110.3 patients (56 patients in the experimental group and 56 in the control group (*N*=112)).

### Recruitment {15}

Patient recruitment will take place in six centres in Belgium: Universitair Ziekenhuis Brussel, Universitair Ziekenhuis Gent, Universitair Ziekenhuis Leuven, AZ Turnhout, Vitaz and Jessa Ziekenhuis. Patients were recruited from April 4, 2022, onwards. Recruitment is expected to last for 2 years with an estimated rate of recruitment of 1 patient per centre per month. Nevertheless, recruitment will continue until the predetermined number of patients is included, regardless of the duration of the recruitment period. Depending on the rate of inclusion, we will contact other centres as well. Treating neurosurgeons or anaesthesiologists (regional coordinating investigator or his/her designee) will inform eligible patients about the project when patients are scheduled for a treatment trajectory with SCS.

## Assignment of interventions: allocation

### Sequence generation {16a}

Patients will be randomly allocated (1:1 ratio) to a personalised rehabilitation programme or usual care using a computer-generated random list. To reduce the predictability of a random sequence, a blocking procedure will be used including random block sizes of 2 and 4 patients. Randomisation will be stratified by investigational site and the duration of sick leave [67] (0 days–1 month, 1 month–1 year, 1–3 years and > 3 years).

### Concealment mechanism {16b}

The study coordinator is an unblinded researcher and is responsible for scheduling all appointments with therapists and patients and, therefore, will have access to the randomisation list (Excel file). The randomisation list is inaccessible for the outcome assessors and statistician. The study coordinator will not perform interventions nor measurements.

### Implementation {16c}

The study coordinator will generate the allocation sequence and is responsible for informing the patients into which group they are randomised. Additionally, the study coordinator will inform the patient about the next steps of the rehabilitation programme, in case the patient is randomised to this group.

## Assignment of interventions: blinding

### Who will be blinded {17a}

The statistician, outcome assessors and treating physicians (neurosurgeons or anaesthesiologists) will be blinded to group allocation. With regard to this, patients will be asked not to communicate with the assessors about the intervention received. Furthermore, at the end of each assessment, the success of assessor blinding will be examined by asking whether the assessor thought the patient had received the experimental or control intervention, including the percentage of certainty (i.e. 50% certainty means a pure guess).

### Procedure for unblinding if needed {17b}

The statistician and outcome assessors will not be unblinded during the trial. In case of a serious worsening of the clinical status of a patient, the treating physician will be unblinded.

## Data collection and management

### Plans for assessment and collection of outcomes {18a}

To optimise the study feasibility and to ensure data protection, the self-reported measures will be completed online using Qualtrics, in the hospital setting. The first assessment is at baseline, the second assessment takes place 6 weeks after IPG implantation, the third assessment immediately after the intervention of 14 weeks, the fourth assessment 3 months after the intervention, the fifth 6 months after the intervention and the sixth at 12 months after the intervention. See Table 1 for an overview of the specific questionnaires for each assessment and Fig. 2 for the participant timeline.

For the functional capacity evaluation, the assessors completed a 2-day training course with Prof. M. Reneman (Rijksuniversiteit Groningen) to ensure a correct performance. As such, all assessors are certified to conduct these evaluations. The results of the functional capacity evaluation will be written down on paper during the assessments. Afterwards, the assessor will enter the data in a specific Excel file for this outcome measurement. A second assessor will control the data entry process.

### Plans to promote participant retention and complete follow-up {18b}

Patients receive a reminder of upcoming appointments and follow-up phone calls to promote participant retention and completion. For patients allocated to the rehabilitation programme, all sessions are scheduled to the patient’s wishes, needs and abilities, in agreement with the schedule of the therapists.

### Data management {19}

Data will be collected via web-based self-reported questionnaires that are provided to the patients on a tablet in the hospital. To avoid missing data, an error message will appear when a question is not filled in. Collected data will include answers to validated questionnaires related to the patients’ ability to work, work status and participation, health-related quality of life, pain intensity, physical activity/functioning, sleep quality, kinesiophobia, healthcare expenditure, anxiety, depression and self-management. In addition, patients will be asked about the duration of their symptoms and general demographic data. Written informed consent of the patients will be collected and provides the basis for the legal ground for the data management.

During the research, the persons responsible for data management and storage will be the predoctoral investigators. Following the research, the PI will be fully responsible for data management and storage. During the research, all obtained data will be stored on a dedicated page on Vrije Universiteit Brussel SharePoint (system-encrypted) with access limited to the investigators and supervisors. A back-up will be foreseen on a secure external hard drive. Following the research, all data will be relocated to the Vrije Universiteit Brussel Archive where it will be archived for 25 years. All possible personal identifiable data (vide infra) will be removed from the archived data.

Personal data will be processed in accordance with the ongoing European Union’s Data Protection Directive and regulation, the relevant Belgian legislation concerning data protection of July 30, 2018, and good clinical practice. As we collect personal identifiable data, the following steps are taken to limit unauthorised access. Informed consents will be preserved at a secure location at the Vrije Universiteit Brussel. Qualtrics (Qualtrics, Provo, UT) will be used for data collection to improve data protection as responses to questionnaires will only be accessible to the investigator. Collected data will be password protected. Personal identifiable and clinical trial data will be separated, with the latter receiving a unique participant ID. Access to informed consents, personal identifiable data and the link with the participant ID will be restricted to the investigators and supervisors and stored separately from the trial data. Eventual further dissemination of data will only occur in a pseudonymised or aggregated way.

The Vrije Universiteit Brussel supports the FOSB metadata standard (= dataset metadata schema defined by the Flemish Open Science Board) which can be mapped to the international DataCite metadata schema. At the project level, the general information (title, investigators, aim, objectives, concepts, hypotheses, funder), protocol, sampling procedure, instruments, hardware and software used to collect data, data handling log, accessibility of the data and data manipulations will be provided in research plans and publications. At the database level, an inventory of the files will be provided in a read-me file. At the data level, a codebook will be provided on how to handle quantitative variables together with the scripts to analyse the data.

### Confidentiality {27}

Participant identification codes will be used to link data to patients. The file containing the link between participant numbers and personal data (i.e. key) will be managed by the researchers and will be locked for access by others. As an additional security measure, the file linking the pseudonymisation to the original direct identifiers will be encrypted before it is uploaded to SharePoint.

### Plans for collection, laboratory evaluation and storage of biological specimens for genetic or molecular analysis in this trial/future use {33}

No biological specimens will be obtained during the conduct of the trial.

## Statistical methods

### Statistical methods for primary and secondary outcomes {20a}

Longitudinal mixed models will be used to evaluate and compare therapy effects. The primary outcome will be analysed as the difference in WAI score from baseline to the short-term primary endpoint immediately after the intervention and to the long-term primary endpoint at 12 months after the intervention (taking into account the longitudinal nature of the trial) between both groups. Baseline variables will be used as covariates. A similar strategy will be applied for secondary outcome measurements. The threshold for statistical significance will be set at *p*<0.05. Statistical as well as clinically significant differences will be defined, and the effect size will be determined.

### Interim analyses {21b}

No interim analyses will be conducted as we do not foresee any potentially serious outcomes.

### Methods for additional analyses (e.g. subgroup analyses) {20b}

Baseline data will provide cross-sectional results on work ability, work status, pain intensity, quality of life, physical activity, sleep quality, kinesiophobia, healthcare expenditure and functioning for the complete PSPS-T2 group and comparisons between possible subgroups. Based on the distribution of the data, group differences at baseline will be assessed using a parametric test or its non-parametric alternative at alpha lower than 0.05. Furthermore, correlation analyses will be performed to unravel correlations between the different outcome measures in patients with PSPS-T2, eligible for SCS. Correlation analysis will be performed with Pearson correlation coefficients if the assumption of a linear relation between two variables is met; otherwise, Spearman correlation coefficients will be calculated and tested at alpha < 0.05. Additionally, the association between self-reporting of the work ability (WAI; primary outcome measure) and objective measurements of work capacity (functional capacity evaluation; secondary outcome measure) will be determined.

Correlation analyses will also be performed to evaluate whether changes in physical and mental ability are related to secondary outcome measures, considering appropriate measures for multiple testing. Furthermore, based on the baseline data, we will try to determine predictive factors and which subgroup of patients will benefit the most from the interventional rehabilitation programme. For this, data obtained at the 12-month assessments will be dichotomised and the baseline variables used as explanatory variables. The predictive value of the secondary outcome measures at baseline for the treatment response at 12 months will be examined.

#### Health economic analysis

First, a within-trial economic evaluation will be conducted. All costs of all patients will be considered, for the time horizon starting from IPG implantation until the end of the follow-up period. Intervention costs will be based on the study notes documenting the duration of each session per patient. The valuation of resource use is based on national tariffs. A societal perspective is adopted as indirect costs of productivity loss are a crucial part in the analyses. Health outcomes will be expressed in two ways. Effects are expressed in percentage physical and mental work ability, which is the primary outcome in this trial. Next, health outcomes will be considered expressed in utility using health state values from the general public. The comparator is usual care (control group). The overall result is expressed in an incremental cost-effectiveness ratio (ICER, i.e. incremental cost divided by the percentage increment in physical and mental work ability and incremental cost divided by the incremental QALY gained). Differences in cost between both groups will be analysed using generalised linear models. A modified Park test will be used to identify the appropriate link function. The point estimates of incremental costs and increment health benefits as described above (deterministic analyses) are subject to uncertainty which will be addressed in probabilistic analyses [68]. We will apply non-parametric bootstrapping to test for statistical differences in costs and health benefits to investigate the uncertainty around these outcomes and summarised in cost-effectiveness acceptability curves indicating the likelihood of the intervention to be cost-effective at any willingness-to-pay threshold. Reporting on the results of the health economic evaluation will be in line with the CHEERS II guidelines [69].

Besides the within-trial health economic evaluation, a model-based evaluation will be conducted in order to estimate the expected costs and health outcomes in both the control and intervention group beyond the follow-up period of the trial. A Markov model will be developed compliant to the commonly used guidelines [70]. We assume a cycle of 1 year in the model and apply a lifetime horizon. Lifetime incremental costs and QALYs will be the input for the ICER calculation. Discount rates of 3% for costs and 1.5% for utilities will be applied, which is in line with the Belgian guidelines [71]. The subsequent probabilistic analyses and reporting strategies are identical to those described above.

### Methods in analysis to handle protocol non-adherence and any statistical methods to handle missing data {20c}

Both intention-to-treat and per-protocol analyses will be conducted to check whether both definitions of the population will point towards similar results, which could demonstrate the robustness of the results. At first, the analysis will be performed on data as observed, since random effects mixed models enable us to conduct the analysis in case one valid measurement is included in the analysis. Hence, intermittent missing is not expected to substantially bias the results. To address possible informative drop-outs, a sensitivity analysis will be performed using multiple imputation, including all available information on background characteristics and outcome. All analysis will be performed in SAS or R.

### Plans to give access to the full protocol, participant-level data and statistical code {31c}

After finalising the project, access restrictions will be applied to the pseudonymised data and will be specified in a data use agreement containing the following elements: evaluation of the re-use request by ethical committee, non-disclosure agreement and warranties for safe storage of data.

## Oversight and monitoring

### Composition of the coordinating centre and trial steering committee {5d}

The Steering Board is the main decision-making and steering body of the project. The Steering Board consists of M.M, L.G., K.P., A.D.S., D.V.D.V. and L.G. The extended Steering Board consists of the Steering Board, together with the project coordinator and three predoctoral students. The Steering Board organised a kick-off meeting at the start of the project to establish common working procedures. All Steering Board members are responsible for the internal communication within their own institution. The main tasks of the Steering Board are (1) agree on common working procedures and management policies, (2) monitor overall progress and follow-up of deliverables, (3) decisions on major changes to the work programme, (4) conflict handling and (5) budget decisions. One of the most important tasks of the Steering Board is also to (re)direct all participants in the implementation of the work programme and the timely achievement of all deliverables. The Extended Steering Board will further assemble meetings at least every 6 months. Additional teleconferences can be organised ad hoc in case of urgent issues. The Extended Steering Board is responsible for assuring the quality of the workflow and project implementation, considering the available resources. The principal investigators in the recruiting centres are responsible for patient recruitment and form the OPERA consortium, together with the Extended Steering Board.

The valorisation board consists of relevant stakeholders (*N*=7) who will be asked to actively contribute to the implementation of the study findings, and any difficulties experienced during the implementation process will be discussed. The researchers are available to support the stakeholders with the implementation process. In addition, the valorisation board will prepare and guide the full utilisation process in the period following the completion of the research project (after-trajectory), together with the Extended Steering Board.

### Composition of the data monitoring committee, its role and reporting structure {21a}

The study coordinator at Vrije Universiteit Brussel will regularly monitor data that are entered in Qualtrics and in the Excel file for the functional capacity evaluation. The study coordinator is independent from the funder of this study and has no competing interests.

### Adverse event reporting and harms {22}

All adverse events (AEs) reported spontaneously by the patient or observed by the assessor will be recorded. Serious adverse events (SAEs) will be reported to the (local) principal investigators as soon as possible, who will be responsible for informing the ethics committee. Reporting suspected unexpected serious adverse reactions (SUSARs) is not applicable, since this study does not include a medicinal product.

### Frequency and plans for auditing trial conduct {23}

The study staff will submit a summary of the progress of the trial to the central ethics committee once a year. Information will be provided on the date of inclusion of the first subject, numbers of subjects included, numbers of subjects that have completed the trial, serious adverse events/serious adverse reactions, other problems and amendments. There is no planned on-site auditing of the trial. However, to ensure compliance with relevant regulations, an independent quality assurance representative may review this study. This implies that auditors will have the right to audit the site(s) at any time during and/or after the completion of the study and will have access to the data generated during the clinical investigation, source documents and patient’s files.

### Plans for communicating important protocol amendments to relevant parties (e.g. trial participants, ethical committees) {25}

All protocol amendments will be approved by the ethics committee prior to implementation. If relevant, patients will be informed of protocol modifications.

### Dissemination plans {31a}

Given the high value of the results for patients, clinicians, society and other stakeholders, the implementation of this rehabilitation programme in clinical settings is the primary goal. All stakeholders will be contacted during the execution of the project to start introducing this rehabilitation programme in relation to PSPS-T2 patients. Regular updates will be provided to all stakeholders to keep everyone informed, involved and motivated for this project. Furthermore, we will communicate findings of this project via the publication of scientific manuscripts and presentations on national and international symposia, as well as through social media. Furthermore, we will write a summary of the main study findings in layman’s terms for patients’ organisations and charities. Additionally, social instances will be contacted, and presentations will be given to make them aware of the study findings.

## Discussion

The OPERA study will evaluate whether a multidisciplinary biopsychosocial approach for improving RTW is different from usual care in terms of work ability for patients with PSPS-T2 after SCS implantation. This allows us to tackle the high burden of patients that are not re-entering the labour market by proceeding towards a personalised multidisciplinary evidence-based conservative intervention for a costly and debilitating condition. If the new intervention, consisting of a personalised biopsychosocial RTW rehabilitation programme, is more effective than the usual care alone for improving work ability in PSPS-T2 patients after SCS implantation, then the new therapy should be applied as the new standard conservative treatment for these patients. Therefore, this project holds the potential to have utilisation goals for several stakeholders among which patients themselves to improve their work ability, health professionals since new treatment guidelines could be developed based on the results of this project and the health security system since the study findings may allow for scientifically evidence-based decision-making, in order to decrease expenses of the public and private health security systems accompanied by PSPS-T2 patients with SCS that are not resuming work.

Furthermore, these study findings could open avenues for similar studies evaluating the benefits of this multidisciplinary programme in patients after failed surgery, where RTW is currently not achieved (i.e. interdisciplinary rehabilitation programme after surgery as such). Hence, the project has the potential to significantly impact upon the progress of science and clinical practice in the field of “failed surgery” (i.e. applicability for other patient groups in whom RTW is not achieved). The awareness is growing that the burden of PSPS-T2 on our society is expected to increase over time due to the annual increase of spinal surgeries [72], but innovative and methodologically rigorous trials exploring the potential to decrease the socio-economic burden are essentially lacking.

### Trial status

Recruitment has started in April 2022 and will be ongoing until 112 patients are included in the study (expected end date December 2023). The current protocol is version 3 of May 2022.

## Data Availability

The Extended Steering Board has access to the pseudonymised final trial dataset. Any materials to support the protocol can be supplied on motivated request, under the condition that the current study is not compromised.

## Abbreviations

EQ5D-5L: EuroQol with five dimensions and 5 levels
FCE: Functional capacity evaluation
HADS: Hospital Anxiety and Depression Scale
HE: Healthcare expenditure
IMBA: Integration von Menschen mit Behinderungen in die Arbeitswelt
ODI: Oswestry disability index
PSPS-T2: Persistent spinal pain syndrome type II
OR: Odds ratio
PAM: Patient activation measure-13
PSQI: Pittsburgh Sleep Quality Index
RTW: Return to work
SCS: Spinal cord stimulation
TSK: Tampa Scale for Kinesiophobia
VAS: Visual analogue scale
WAI: Work Ability Index
WORQ: Work Rehabilitation Questionnaire
